# Supplementary material for: Exploring the Use of Hydroxytyrosol and Some of Its Esters in Food-Grade Nanoemulsions: Establishing Connection between Structure and Efficiency
Source: Antioxidants (Basel). 2023 Nov 14;12(11):2002. doi: 10.3390/antiox12112002 (PMC10669426; doi:10.3390/antiox12112002)
Supplement: Supplementary file 1 [file antioxidants-12-02002-s001.zip › antioxidants-2705704-supplementary.pdf]

1S. ^{1}H NMR and ^{13}C NMR values for the HT derivatives.

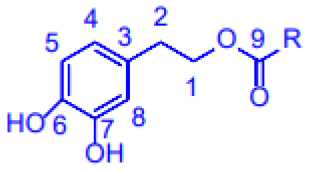

C_1, R = -H
C_{n+1} R = $-(CH_2)_{n-1}CH_3$'

C1. ^{1}H NMR (400 MHz, acetone D6): δ 7,7 (1H, H-9); 6,76 (d, J = 8,0 Hz, 1H, H-5); 6,75 (d, J = 8 Hz, 1H, H-8); 6,59 (dd, 3J = 8,0 Hz, 4J =2,0 Hz, 1H, H-4); 4,27 (t, J = 8,0 Hz, 2H, H-1); 2,81 (t, J = 8,0 Hz, 2H, H-2).

^{13}C NMR (100 MHz, acetone D6): δ 161,9 (C-9); 145,9 (C-7); 144,6 (C-6); 130,2 (C-3); 121,1 (C-4); 116,8 (C-5); 116,1 (C-8); 65,2 (C-1); 35,0 (C-2).

C2. ^{1}H NMR (400 MHz, acetone D6): δ 6,75 (d, J = 8,0 Hz, 1H, H-5); 6,74 (d, J = 8,0 Hz, 1H, H-8); 6,58 (dd, 3J = 8,0 Hz, 4J =2,0 Hz, 1H, H-4); 4,15 (t, J = 8,0 Hz, 2H, H-1); 2,76 (t, J = 8,0 Hz, 2H, H2); 1,96 (t, J = 8,0 Hz, 3H, H-10).

^{13}C NMR (100 MHz, acetone D6): δ 170,0 (C-9); 145,0 (C-7); 143,6 (C-6); 129,7 (C-3); 120,1 (C-4); 115,9 (C-5); 115,2 (C-8); 64,8 (C-1); 34,2 (C-2); 27,0 (C-10).

C3. ^{1}H NMR (400 MHz, acetone D6): δ 6,76 (d, J = 8,0 Hz, 1H, H-5); 6,75 (d, J = 8,0 Hz, 1H, H-8); 6,58 (dd, 3 J = 8,0 Hz, 4 J =2,0 Hz, 1H, H-4); 4,18 (t, J = 8,0 Hz, 2H, H-1); 2,77 (t, J = 8,0 Hz, 2H, H2); 2,28 (quart, J = 8,0 Hz, 2H, H-10); 1,05 (t, J = 8,0 Hz, 3H, H-11).

^{13}C NMR (100 MHz, acetone D6): δ 173,5 (C-9); 145,0 (C-7); 143,6 (C-6); 129,7 (C-3); 120,2 (C-4); 116,0 (C-5); 115,2 (C-8); 64,8 (C-1); 34,3 (C-2); 27,0 (C-10); 8,5 (C-11).

C4. ^{1}H NMR (400 MHz, acetone D6): δ 6,76 (d, J = 8,0 Hz, 1H, H-5); 6,75 (d, J = 8,0 Hz, 1H, H-8); 6,58 (dd, 3J = 8,0 Hz, 4J =2.0 Hz, 1H, H-4); 4,18 (t, J = 8,0 Hz, 2H, H-1); 2;76 (t, J = 8,0 Hz, 2H, H2); 2,24 (t, J = 8,0 Hz, 2H, H-10); 1,58 (sext, J = 8,0 Hz, 2H, H-11); 0,89 (t, J = 8,0 Hz, 3H, H-12).

^{13}C NMR (100 MHz, acetone D6): δ 173,5 (C-9); 145,9 (C-7); 144,5 (C-6); 130,6 (C-3); 121,0 (C4); 116,8 (C-5); 116,0 (C-8); 65,6 (C-1); 36,5 (C-2); 35,2 (C-10); 19,0 (C-11); 13,8 (C-12).

C6. ^{1}H NMR (400 MHz, acetone D6): δ 6,75 (d, J = 8,0 Hz, 1H, H-5); 6,74 (d, J = 8,0 Hz, 1H, H-8); 6,58 (dd, 3J = 8,0 Hz, 4J =2,0 Hz, 1H, H-4); 4,18 (t, J = 8,0 Hz, 2H, H-1); 2,77 (t, J = 8,0 Hz, 2H, H-7 2); 2,26 (t, J = 8,0 Hz, 2H, H-10); 1,57 (quint, J = 8,0 Hz, 2H, H-11); 1,28 (m, 4H, H-12); 0,88 (t, J =8,0 Hz, 3H, H-13).

^{13}C NMR (100 MHz, acetone D6): δ 172,8 (C-9); 145,0 (C-7); 143,6 (C-6); 129,7 (C-3); 120,1 (C 4); 115,9 (C-5); 115,2 (C-8); 64,7 (C-1); 34,3 (C-2); 33,7 (C-10); 31,1 (C-11); 24,5 (C12); 22,1 (C 13); 13,3 (C-14).

C8. ^{1}H NMR (400 MHz, acetone D6): δ 6,75 (d, J = 8,0 Hz, 1H, H-5); 6,74 (d, J = 8,0 Hz, 1H, H-8);

6,58 (dd, 3J = 8,0 Hz, 4J =2,0 Hz, 1H, H-4); 4,17 (t, J = 8,0 Hz, 2H, H-1); 2,77 (t, J = 8,0 Hz, 2H, H2); 2,26 (t, J = 8,0 Hz, 2H, H-10); 1,57 (quint, J = 8,0 Hz, 2H, H-11); 1,28 (m, 6H, H-12); 0,88 (t, J =8,0 Hz, 3H, H-13).

^{13}C NMR (100 MHz, acetone D6): δ 172,7 (C-9); 145,0 (C-7); 143,9 (C-6); 129,7 (C-3); 120,1 (C-4); 115,8 (C-5); 115,2 (C-8); 64,7 (C-1); 34,3 (C-2); 33,8 (C-10); 31,5 (C-11); 24,8 (C12); 24,8 (C13); 22,4 (C-14); 22,4 (C-15); 13,4 (C-16).

C10. ^{1}H NMR (400 MHz, acetone D6): δ 6,75 (d, J = 8,0 Hz, 1H, H-5); 6,74 (d, J = 8,0 Hz, 1H, H-8);

6,58 (dd, 3J = 8,0 Hz, 4J =2,0 Hz, 1H, H-4); 4,18 (t, J = 8,0 Hz, 2H, H-1); 2,77 (t, J = 8,0 Hz, 2H, H2); 2,28 (t, J = 8,0 Hz, 2H, H-10); 1,57 (quint, J = 8,0 Hz, 2H, H-11); 1,29 (m, 12H, H-12); 0,88 (t, J= 8,0 Hz, 3H, H-13). 13C NMR (100 MHz, acetone D6): δ 172,7 (C-9); 145,0 (C-7); 143,6 (C-6); 129,7 (C-3); 120,3 (C-4); 115,9 (C-5); 115,1 (C-8); 64,6 (C-1); 34,3 (C-2); 33,8 (C-10); 31,7 (C-11); 24,8 (C12); 24,8 (C13); 24,8 (C14); 22,4 (C-15); 22,4 (C-16); 22,4 (C-17); 13,5 (C-18).

C12. ^{1}H NMR (400 MHz, acetone D6): δ 6,75 (d, J = 8,0 Hz, 1H, H-5); 6,74 (d, J = 8,0 Hz, 1H, H-8);6,58 (dd, 3J = 8,0 Hz, 4J =2,0 Hz, 1H, H-4); 4,17 (t, J = 8,0 Hz, 2H, H-1); 2,77 (t, J = 8,0 Hz, 2H, H2), 2,26 (t, J = 8,0 Hz, 2H, H-10); 1,57 (quint, J = 8,0 Hz, 2H, H-11), 1,29 (m, 16H, H-12); 0,88 (t, J= 8,0 Hz, 3H, H-13).

^{13}C NMR (100 MHz, acetone D6): δ 172,7 (C-9); 145,0 (C-7); 143,6 (C-6); 129,7 (C-3); 120,1 (C-4); 115,8 (C-5); 115,2 (C-8); 64,7 (C-1); 34,3 (C-2); 33,8 (C-10); 31,7 (C-11); 24,8 (C12); 24,8 (C13); 24,8 (C14); 24,8 (C15); 22,4 (C-16); 22,4 (C-17); 22,4 (C-18); 22,4 (C-19); 13,5 (C-20).

C16. ^{1}H NMR (400 MHz, acetone D6): δ 6,75 (d, J = 8,0 Hz, 1H, H-5); 6,74 (d, J = 8,0 Hz, 1H, H-8); 6,58 (dd, 3J = 8,0 Hz, 4J =2,0 Hz, 1H, H-4); 4,17 (t, J = 8,0 Hz, 2H, H-1); 2,76 (t, J = 8.0 Hz, 2H, H2); 2,26 (t, J = 8,0 Hz, 2H, H-10); 1,57 (quint, J = 8,0 Hz, 2H, H-11); 1,29 (m, 24H, H-12); 0,88 (t, J= 8,0 Hz, 3H, H-13).

^{13}C NMR (100 MHz, acetone D6): δ 173,6 (C-9); 145,9 (C-7); 144,5 (C-6); 130,6 (C-3); 122,0 (C-4); 116,8 (C-5); 116,0 (C-8); 65,6 (C-1); 35,2 (C-2); 34,7 (C-10); 32,7 (C-11); 25,7 (C12); 25,7 (C13); 25,7 (C14); 25,7 (C15); 25,7 (C16); 25,7 (C17); 23,3 (C-18); 23,3 (C-19); 23,3 (C-20); 23,3 (C-21); 23,3 (C-22); 23,3 (C-23); 14,4 (C-24).
